# Multimodality imaging diagnosis of a left ventricular pseudoaneurysm causing dynamic coronary compression

**DOI:** 10.1093/ehjcr/ytag362

**Published:** 2026-05-13

**Authors:** Bernardo Resende, Tomás Carlos, João Rosa, Lino Gonçalves

**Affiliations:** Serviço de Cardiologia, Unidade Local de Saúde de Coimbra, Praceta Professor Mota Pinto, Celas, Coimbra 3004-561, Portugal; Serviço de Cardiologia, Unidade Local de Saúde de Coimbra, Praceta Professor Mota Pinto, Celas, Coimbra 3004-561, Portugal; Serviço de Cardiologia, Unidade Local de Saúde de Coimbra, Praceta Professor Mota Pinto, Celas, Coimbra 3004-561, Portugal; Faculdade de Medicina da Universidade de Coimbra, Coimbra 3000-548, Portugal; Coimbra Institute for Biomedical Imaging and Translational Research (CIBIT), Coimbra 3000-548, Portugal; Serviço de Cardiologia, Unidade Local de Saúde de Coimbra, Praceta Professor Mota Pinto, Celas, Coimbra 3004-561, Portugal; Faculdade de Medicina da Universidade de Coimbra, Coimbra 3000-548, Portugal; Coimbra Institute for Clinical and Biomedical Research (iCBR), Coimbra 3000-548, Portugal

**Keywords:** Multimodality Cardiac Imaging, Dynamic Coronary Compression, Left Ventricular Pseudoaneurysm

## Case description

An 85-year-old man with a history of surgical aortic valve replacement in 2018, with concomitant transannular aortic root enlargement and septal myectomy, had a postoperative course complicated by hypovolemic shock requiring surgical re-exploration. In April 2025, he presented to the emergency department with a three-month history of exertional angina.

**Figure 1:**
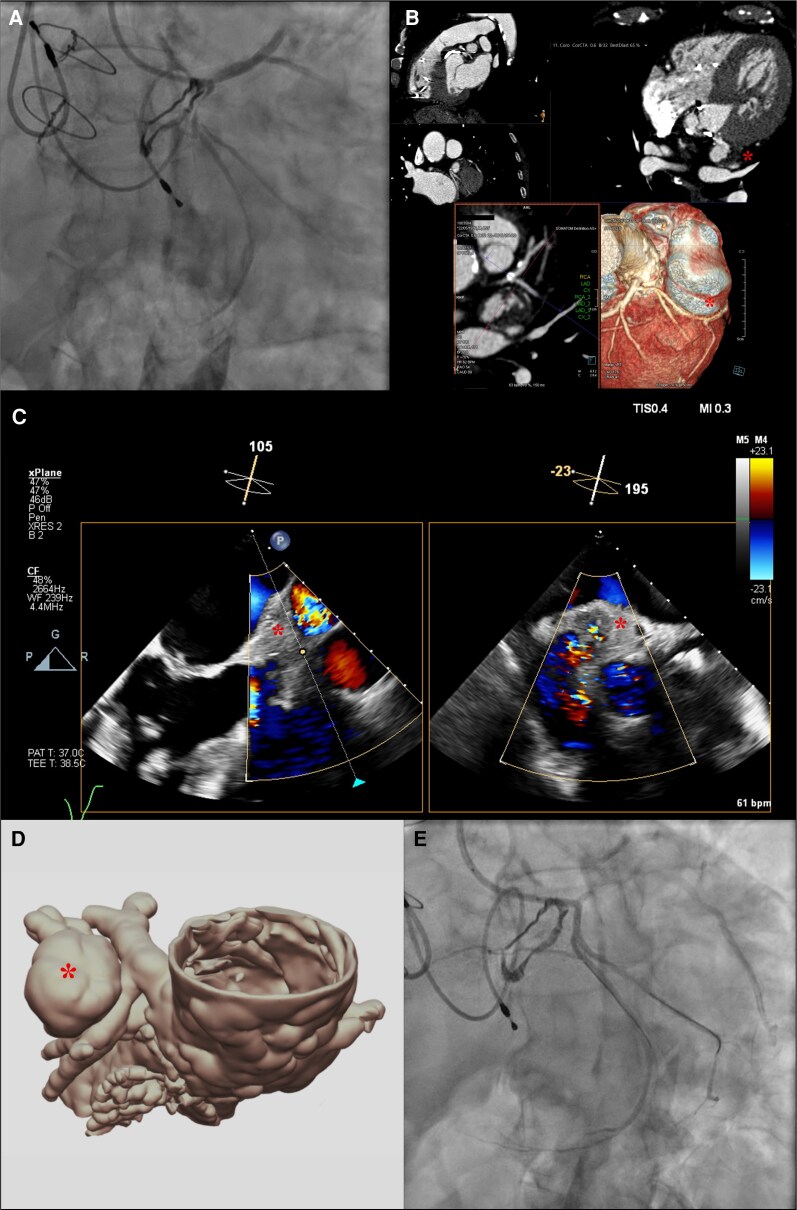
Multimodality imaging approach to a left ventricular outflow tract (LVOT) lesion (*). (*A*) Initial coronary angiography demonstrating luminal narrowing of the circumflex artery; (*B*) Axial view of coronary computed tomography angiography (CTA) illustrating the spatial relationship between the lesion (*) and the circumflex artery and 3D volume-rendered reconstruction from coronary CTA; (*C*) Transoesophageal echocardiography (TEE), 105° view, showing the lesion (*) *in close proximity to the left ventricular cavity and 195°/−23° view, confirming the presence and extent of the lesion* (*); (*D*) Patient-specific 3D-printed anatomical model; (*E*) Final angiographic result following implantation of a drug-eluting stent in the circumflex artery.

Coronary angiography excluded obstructive epicardial coronary artery disease but revealed dynamic extrinsic compression of the circumflex artery (*Figure 1A*, Supplementary material online, *Video S1*). Subsequent evaluation with cardiac computed tomography (CT) angiography (*Figure 1B*) and transoesophageal echocardiography (*Figure 1C*, Supplementary material online, *Video S2*) identified a partially calcified, Doppler flow-filled mass (23 × 32 mm) adjacent to the left ventricular outflow tract, not present on preoperative imaging in 2018. During hospitalization, serial blood cultures remained negative, and 18F-FDG positron emission tomography demonstrated no pathological uptake in the region of the lesion or prosthetic valve. A contained left ventricular rupture with pseudoaneurysm formation was deemed the most likely diagnosis, representing a rare late complication of cardiac surgery.

The case was discussed by the heart team, and surgery was considered high-risk. Percutaneous closure using patient-specific 3D-printed modelling (*Figure 1D*) was attempted but unsuccessful. Given the persistence of angina, percutaneous coronary intervention with drug-eluting stent implantation (2.0 × 18 mm) in the circumflex artery was performed, accompanied by optimal medical therapy (*Figure 1E*, Supplementary material online, *Video S3*). The patient experienced complete symptom resolution, with no adverse events at 6-month follow-up.

The present report highlights the role of multimodality imaging in the diagnosis and procedural planning of complex structural complications. In other clinical scenarios, when appropriate, intravascular ultrasound may offer additional insight into coronary compression, while cardiac magnetic resonance may provide further tissue characterization. Although definitive surgical correction would presumably have offered the most complete treatment, tailoring management to the patient’s condition rendered percutaneous coronary intervention a viable and effective option for symptom control.

## Supplementary Material

ytag362_Supplementary_Data

## Data Availability

The data underlying this article are available from the corresponding author upon reasonable request.

## Lead author biography

Bernardo Resende is a Cardiology Resident at the Unidade Local de Saúde de Coimbra, Portugal. He obtained his Integrated Master’s Degree in Medicine from NOVA Medical School, Lisbon. His clinical interests include acute coronary syndromes, heart failure, cardiovascular imaging, and preventive cardiology. He is actively involved in clinical research, with publications in peer-reviewed journals and participation as sub-investigator in international randomized clinical trials and multicentre observational studies. Bernardo is a member of the Portuguese Society of Cardiology and several European Society of Cardiology associations, and currently serves as a peer reviewer for international medical journals.

## Notes

**Consent:** A written informed consent was obtained from the patient to publish these images in accordance with national legislation.

